# Iron uptake by ZIP8 and ZIP14 in human proximal tubular epithelial cells

**DOI:** 10.1007/s10534-019-00183-7

**Published:** 2019-02-26

**Authors:** S. E. G. van Raaij, S. K. S. Srai, D. W. Swinkels, R. P. L. van Swelm

**Affiliations:** 1 Department of Laboratory Medicine, Radboud Institute for Molecular Life Sciences, Radboud University Medical Center, Nijmegen, The Netherlands; 2 Division of Biosciences, Department of Structural & Molecular Biology, University College London, London, UK; 3 Department of Laboratory Medicine, Translational Metabolic Laboratory (830), Radboud University Medical Center, P.O. Box 9101, 6500 HB Nijmegen, The Netherlands

**Keywords:** Iron, Transferrin, Non-transferrin-bound iron, Proximal tubular epithelial cell, ZIP8, ZIP14

## Abstract

In patients with iron overload disorders, increasing number of reports of renal dysfunction and renal iron deposition support an association between increased iron exposure and renal injury. In systemic iron overload, elevated circulating levels of transferrin-bound (TBI) and non-transferrin-bound iron (NTBI) are filtered to the renal proximal tubules, where they may cause injury. However, the mechanisms of tubular iron handling remain elusive. To unravel molecular renal proximal tubular NTBI and TBI handling, human conditionally immortalized proximal tubular epithelial cells (ciPTECs) were incubated with ^55^Fe as NTBI and fluorescently labeled holo-transferrin as TBI. Ferrous iron importers ZIP8 and ZIP14 were localized in the ciPTEC plasma membrane. Whereas silencing of either ZIP8 or ZIP14 alone did not affect ^55^Fe uptake, combined silencing significantly reduced ^55^Fe uptake compared to control (p < 0.05). Furthermore, transferrin receptor 1 (TfR1) and ZIP14, but not ZIP8, colocalized with early endosome antigen 1 (EEA1). TfR1 and ZIP14 also colocalized with uptake of fluorescently labeled transferrin. Furthermore, ZIP14 silencing decreased ^55^Fe uptake after ^55^Fe-Transferrin exposure (p < 0.05), suggesting ZIP14 could be involved in early endosomal transport of TBI-derived iron into the cytosol. Our data suggest that human proximal tubular epithelial cells take up TBI and NTBI, where ZIP8 and ZIP14 are both involved in NTBI uptake, but ZIP14, not ZIP8, mediates TBI-derived iron uptake. This knowledge provides more insights in the mechanisms of renal iron handling and suggests that ZIP8 and ZIP14 could be potential targets for limiting renal iron reabsorption and enhancing urinary iron excretion in systemic iron overload disorders.

**Electronic supplementary material:**

The online version of this article (10.1007/s10534-019-00183-7) contains supplementary material, which is available to authorized users.

## Introduction

Iron is an essential element for life, but it can also be harmful by catalyzing the formation of reactive oxygen species in the Fenton reaction (Koppenol 1993). The human body is able to regulate iron uptake and storage, but has limited abilities to regulate iron excretion (Brissot and Loreal 2016). Therefore, disturbed intestinal iron uptake in hereditary hemochromatosis, and frequent red blood cell transfusions in β-thalassemia major, can result in systemic iron overload and organ damage (Brissot and Loreal 2016). In these disorders, patients present with increased levels of iron bound to the circulating transport protein transferrin (transferrin-bound iron; TBI) (Brissot and Loreal 2016). Once transferrin is largely saturated, non-transferrin-bound iron (NTBI) can be detected (Brissot et al. 2012; de Swart et al. 2016). In contrast to iron bound to transferrin, iron in NTBI is only loosely bound to molecules such as citrate, available for redox cycling and, therefore, considered a toxic iron species (Brissot et al. 2012; Cabantchik 2014). Nowadays, iron-removal therapies decrease hepatic and cardiac iron loading mortality and extend patient’s lives (Adams and Barton 2010; Adams et al. 1991; Brittenham 2011; Niederau et al. 1996). However, in recent years, kidney function abnormalities have been arising in patients with β-thalassemia major (Bhandari and Galanello 2012), including increased urinary excretion of *N*-acetyl-d-glucosaminidase (NAG) and β-2-microglobulin, indicators of renal proximal tubular damage (Aldudak et al. 2000; Hashemieh et al. 2017; Koliakos et al. 2003; Mohkam et al. 2008; Smolkin et al. 2008; Sumboonnanonda et al. 1998). Also, renal iron deposition has been observed in adult patients with hereditary hemochromatosis (Chmieliauskas et al. 2017; Marble and Bailey 1951; Okumura et al. 2002; Ozkurt et al. 2014; Rous 1918) or β-thalassemia syndromes (Hashemieh et al. 2017; Landing et al. 1989; Ong-ajyooth et al. 1998). In vitro studies have shown that iron exposure can result in decreased cellular viability in murine and human renal tubular epithelial cells (Sheerin et al. 1999; Sponsel et al. 1996; Zager and Burkhart 1997). Altogether, these findings support an association between increased renal tubular iron exposure and renal tubular injury in systemic iron overload.

Tubular epithelial cells in the kidney share many iron handling proteins with other organ systems, such as transferrin receptor 1 (TfR1), divalent metal transporters ZIP8 (SLC39A8), ZIP14 (SLC39A14) and divalent metal transporter 1 (DMT1, SLC11A2), and iron exporter ferroportin, but their precise cellular localization and function in renal iron handling remain uncertain (Martines et al. 2013; Smith and Thevenod 2009; Thevenod and Wolff 2016). Circulating TBI is suggested to be filtered into the tubular lumen by the glomerulus (Norden et al. 2001; Zhang et al. 2007). Subsequently, iron in the tubular lumen has been reported to be completely reabsorbed by endocytic transport in renal tubular cells (Thevenod and Wolff 2016). In agreement, hardly any iron is found in urine of healthy volunteers (Green et al. 1968; Rodriguez and Diaz 1995). TBI reabsorption has been reported to mainly take place in proximal tubular epithelial cells (PTs), facilitated by TfR1 and the megalin–cubilin receptor complex (Kozyraki et al. 2001; Norden et al. 2001; Ohno et al. 2005; Zhang et al. 2007). After dissociation from transferrin, iron transport from the endosome into the cytosol is suggested to involve a divalent metal transporter. Although not yet clarified in PTs, studies in other cells suggest a role for ZIP8, ZIP14 and/or DMT1 in this process (Ji and Kosman 2015; Shindo et al. 2006; Zhao et al. 2010). In the cytosol, iron is oxidized by H-ferritin and stored in L-ferritin, may be utilized by iron requiring processes or exported into the blood stream by the cellular exporter ferroportin (Thevenod and Wolff 2016). Tubular NTBI could be directly derived from the circulation by glomerular filtration or dissociated from filtered TBI as a result of acidification of the filtrate passing along the nephron (Martines et al. 2013; Moulouel et al. 2013; Thevenod and Wolff 2016). In vitro studies have reported that ZIP8 and ZIP14 are involved in direct NTBI uptake from the plasma membrane (Coffey and Knutson 2017; Ji and Kosman 2015; Liuzzi et al. 2006; Pinilla-Tenas et al. 2011; Wang et al. 2012), but evidence for PTs is lacking.

In this study, we first characterized TBI and NTBI handling in human conditionally immortalized proximal tubular epithelial cells (ciPTECs). Human ciPTECs, originating from renal tissue, have previously been shown to express functional influx and efflux transporters for solute reabsorption and drug excretion (Jansen et al. 2014; Wilmer et al. 2010). Next, we examined the localization of divalent metal transporters ZIP8, ZIP14 and DMT1 in these cells, i.e. at the plasma membrane and/or in endosomes, and studied the role of ZIP8 and ZIP14 in mediating iron uptake from NTBI and TBI.

## Materials and methods

### Cell culture

ciPTECs (clone T1), kindly provided by the department of Pharmacology and Toxicology, Radboud Institute for Molecular Life Sciences, Radboud University Medical Center, Nijmegen (Jansen et al. 2014), were cultured using DMEM HAM’s F-12 phenol red-free medium (Thermo Fisher Scientific) containing 5 μg/ml insulin, 5 μg/ml transferrin, 5 ng/ml selenium, 36 ng/ml hydrocortisone, 10 ng/ml epithelial growth factor 40 pg/ml tri-iodothyronine (all Sigma Aldrich), 10% (v/v) fetal calf serum (FCS; Greiner Bio-one), and 1% (v/v) penicillin–streptomycin (Thermo Fisher Scientific). Cells were cultured at 33 °C and 5% CO_2_ and maturated for 24 h at 33 °C and 5% CO_2_ and 7 days at 37 °C and 5% CO_2_ prior to experiments. Cells were cultured on transwell permeable supports to obtain polarization [Corning^®^ Transwell^®^, coated with 50 µg/ml collagen VI (both Sigma Aldrich)] for ZIP8, ZIP14, DMT1 and ferroportin immunostaining, ^55^Fe transport and Alexa Fluor 546-conjugated human holotransferrin (Alexa546-Transferrin, Thermo Fisher Scientific) uptake studies. Transepithelial electrical resistance (TEER) measurements and FITC-inulin permeability analysis were performed as previously described (Jansen et al. 2014).

### Iron exposure

To simulate NTBI exposure, cells were exposed to 0–500 µM ferric citrate (FeC, Sigma Aldrich). FeC was dissolved overnight in MQ water at 37 °C and added to medium devoid of FCS. Cell pellets were collected and stored at − 80 °C prior to iron assessment or immunoblotting. For TBI uptake studies, cells were depleted of transferrin by incubation in DMEM HAM’s F-12 medium without supplements for 2 h and exposed to 25 µg/ml Alexa546-Transferrin for 30 min. In iron loading conditions, cells were instead exposed to 100 µM FeC and 100 µg/ml holo-transferrin (Sigma Aldrich) for 2 h and subsequently exposed to Alexa546-Transferrin. Afterwards, cells were used for immunostaining as described below. To simulate iron overload exposure, cells were exposed to 100 µM FeC in medium supplemented with FCS for 48 h and used for immunostaining of ZIP8 and ZIP14.

### Small interfering RNAs (siRNAs)

Cells were seeded at 20% confluency and transfected on two consecutive days with 50 pmol (ZIP8, ZIP14) or 100 pmol (ferroportin) small interfering RNAs (siRNAs) (ON-TARGETplus SMARTpool siRNAs for SLC39A8, SLC39A14, SLC40A1 and non-targeting pool as scrambled control, Dharmacon) and 5 µl oligofectamine in Opti-MEM (both Thermo Fisher Scientific). After 4 h, fresh medium was added. Cells were analyzed 48 h after the second transfection.

### Protein isolation and immunoblotting

Proteins were isolated using RIPA buffer [0.15 M NaCl, 0.012 M Sodium Deoxycholate, 1% NP40, 0.1% SDS, 0.05 M Tris, pH 7.5, freshly supplemented with protease inhibitors (Roche Complete Mini, Roche)]. Protein concentration was determined using the Pierce BCA assay kit according to the manufacturer’s instructions (Thermo Fisher Scientific). Protein samples were prepared in loading buffer, separated on SDS-PAGE gels, transferred to a nitrocellulose or PVDF membrane and incubated with primary antibody overnight at 4 °C. After 1 h incubation at RT with secondary antibody, proteins were visualized on an Odyssey fluorescence scanner (total ferritin, β-actin) or LAS-3000 scanner for chemiluminescence (all other primary antibodies). Antibodies and dilutions are summarized in Online Resource 1.

### Cell surface biotinylation

Cells were biotinylated with 0.5 mg/ml Sulfo-NHS-LC-LC-biotin (Thermo Fisher Scientific) for 30 min at 4 °C with gentle shaking. Protein lysates were collected by scraping and incubated overnight with Neutravidin beads (Thermo Fisher Scientific) to isolate cell surface biotinylated proteins. After eluting cell surface proteins from the beads in Laemmli buffer (Biorad) supplemented with 50 mM DTT for 30 min at 37 °C, proteins were directly used for immunoblotting since the Pierce BCA assay could not be applied in this buffer solution. Therefore, both membrane protein and total lysate protein fraction were loaded on SDS-PAGE gels to obtain comparable Na K ATPase bands and allow comparison of proteins of interest between the two fractions.

### RNA isolation and quantitative PCR

RNA isolation was performed using TRIzol (Thermo Fisher Scientific) according to the manufacturer’s instructions. A reverse transcription reaction was performed with 1 μg RNA, 4 μl first strand buffer, 1 μl dNTPs (12.5 mM), 2.04 μl random primers, 2 μl DDT, 1 μl M-MLV (all Thermo Scientific) and 0.5 μl RNAsin (Promega Corporation). The PCR cycle consisted of 10 min at 20 °C, 45 min at 42 °C and 10 min at 95 °C. Quantitative PCR was performed on a CFX96 (Bio-rad) using 4 µl cDNA (10 ng/ml), 10 µl SYBR Green Power master mix (Applied Biosystems) and 6 µl primer mix (containing 1 µM forward primer and reverse primer). The PCR protocol was as follows: 7 min at 95 °C, 40 cycles of 15 s at 95 °C and 1 min at 60 °C, and 10 min at 95 °C, with a measurement at the end of each cycle. Fold change values were calculated using the ΔΔCt formula. Primers are summarized in Online Resource 2.

### Immunofluorescent staining

Cells seeded on coverslips or transwell supports were fixed with 4% paraformaldehyde or 2% paraformaldehyde supplemented with 4% sucrose, permeabilized with 0.5% Triton X-100, 0.2% Tween-20 or 1% SDS and incubated with primary antibody overnight at 4 °C. Subsequently, cells were stained with fluorescent secondary antibody for 1 h at RT and counterstained with DAPI (4′,6-diamidino-2-phenylindole, 300 µM, Thermo Fisher Scientific). Images were taken using the Zeiss S/N 3834004266 or confocal Olympus FV1000. Co-stainings were performed for TfR1, ZIP8, ZIP14 and DMT1 with early endosome antigen 1 (EEA1). Antibodies are summarized in Online Resource 1.

### Iron assessment

Intracellular iron levels were determined using the chromogen bathophenanthroline as described (Torrance and Bothwell 1968). Iron concentrations were calculated by comparison to a standard curve of ferrous sulphate and corrected for protein concentration.

### ^55^Fe transport

Cells were incubated with 0.2 µM ^55^FeCl_3_ (Perkin Elmer) in a mixture with 100 µM FeC and 1 mM ascorbic acid (Sigma Aldrich) in Krebs–Henseleit-HEPES (KH-H) buffer supplemented with 2.5 mM CaCl_2_·2H_2_O, 25 mM NaHCO_3_ and 10 mM HEPES pH 7.4 (Sigma Aldrich). For cells grown in transwell supports, cells were depleted of iron in DMEM HAM’s F-12 medium without supplements for 2 h and incubated with ^55^Fe in KH-H in the apical compartment or in medium in the basolateral compartment for 8 h at 37 °C. For ZIP8 and ZIP14 siRNA experiments, cells were depleted of iron in KH-H buffer for 2 h and incubated with ^55^Fe for 30 min afterwards. For ferroportin siRNAs, cells were exposed to ^55^Fe for 2 h. Subsequently, cells were washed with ice-cold KH-H and harvested using RIPA buffer. Afterwards, radioactivity was measured using liquid scintillation counting. Radioactivity in protein pellets was corrected for protein concentration.

^55^Fe-TBI was prepared by incubating apo-transferrin (Sigma Aldrich) with 7.5 nmol ^55^FeCl_3_ and 75 nmol sodium citrate in KH-H for 1 h at RT. Unbound iron was removed by repeated washing in 30 K Amicon filter units. After iron depletion in KH-H buffer for 2 h, cells were exposed to 2 μM ^55^Fe-TBI for 4 h and analyzed as described above.

### Statistical analysis

Data were statistically analyzed using GraphPad Prism and presented as mean ± SEM. Results were analyzed by one-way ANOVA with Dunnett’s post test or Student’s *t* test where appropriate. Differences were considered statistically significant when p < 0.05.

## Results

### Characterization ciPTEC model for studying iron handling

**Fig. 1:**
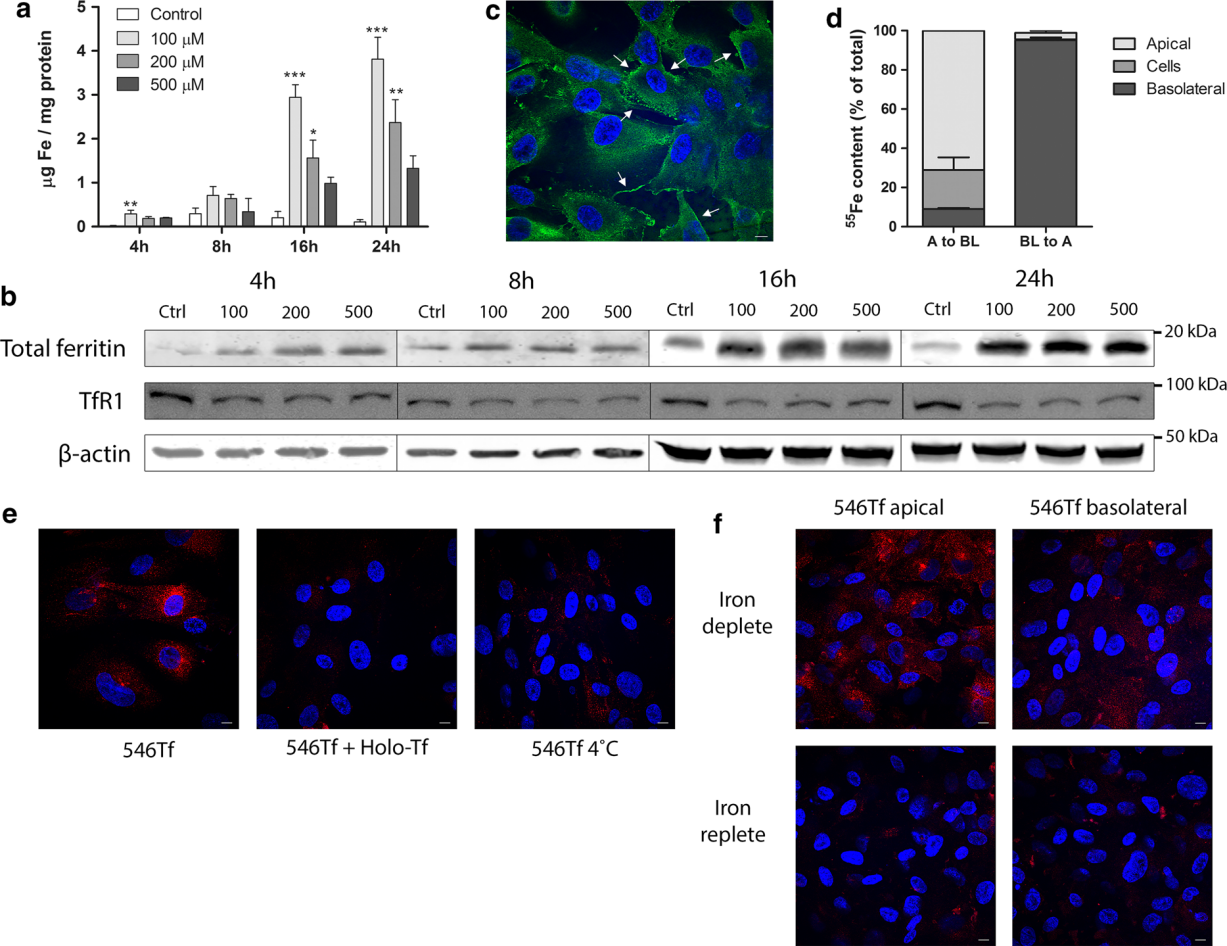
Uptake of non-transferrin-bound iron and transferrin-bound iron in ciPTECs. Intracellular iron concentration (**a**), total ferritin and transferrin receptor 1 (TfR1) protein levels (**b**) in ciPTECs after incubation with different concentrations of ferric citrate (NTBI). Zona occludens 1 (ZO-1) immunostaining (in green) confirming monolayer integrity in polarized ciPTECs. ZO-1 tight junctions indicated by arrows (**c**). ^55^Fe content in apical compartment, basolateral compartment and cell lysate after ^55^Fe exposure from the apical (A to BL) or basolateral (BL to A) cellular side (**d**). Alexa546-Transferrin (546Tf, in red) internalization alone, combined with holo-transferrin (Holo-Tf) or at 4 °C (**e**). 546Tf internalization in iron deplete or iron replete conditions, from apical or basolateral cellular side (**f**). Nuclei counterstained with DAPI (in blue). Representative images and graphs showing mean of three independent experiments for each time point or FeC concentration except n = 6 for panel c. Scale bar 5 µM. One-way ANOVA with Dunnett’s post test compared to control at each time point was used in **a**; *p < 0.05; **p < 0.01; ***p < 0.001. (Color figure online)

We first characterized the presence and abundance of known iron handling proteins in ciPTECs in unstimulated conditions and after iron exposure. Upon exposure to NTBI (FeC), ciPTECs showed a time-dependent increase in intracellular iron levels, which was statistically significant for 100 and 200 µM FeC after exposure for 16 and 24 h (Fig. 1a). This was complemented by increased total ferritin and decreased TfR1 protein levels compared to control (Fig. 1b), confirming genomic iron responsive element – iron responsive protein (IRE-IRP) regulation of these proteins in ciPTECs (Muckenthaler et al. 2017). Next, we examined iron uptake in polarized ciPTECs, grown on transwell supports. Monolayer integrity was confirmed by minimal paracellular permeability of the diffusion marker FITC-inulin (7.6 ± 0.8 pmol/min/cm^2^), appropriate transepithelial electric resistance (TEER; ≥ 140 Ω/cm^2^) and clear expression of the tight junction protein zona occludens 1 (ZO-1; Fig. 1c) (Jansen et al. 2014). In ciPTECs cultured on transwell supports, apical ^55^Fe exposure resulted in intracellular iron loading while basolateral ^55^Fe exposure showed only limited cellular uptake (Fig. 1d). This indicates that ciPTECs take up iron mainly from the apical cellular side. Additionally, we characterized the direction of iron export in ciPTECs. Polarized ciPTECs showed basolateral iron export after apical ^55^Fe exposure, while basolateral ^55^Fe exposure showed negligible apical iron export (Fig. 1d). CiPTECs also demonstrated uptake of Alexa546-Transferrin (holo-transferrin), which was diminished in combination with unlabelled holo-transferrin, indicating ligand-specific competition, or at 4 °C, suggesting active uptake (Fig. 1e). Alexa546-Transferrin uptake was mostly observed after apical exposure, and little uptake was seen after basolateral exposure, whereas both apical and basolateral uptake was reduced in iron-loaded cells (Fig. 1f). The latter suggests that ciPTECs regulate TBI uptake based on intracellular iron levels through IRE-IRP regulation.

**Fig. 2:**
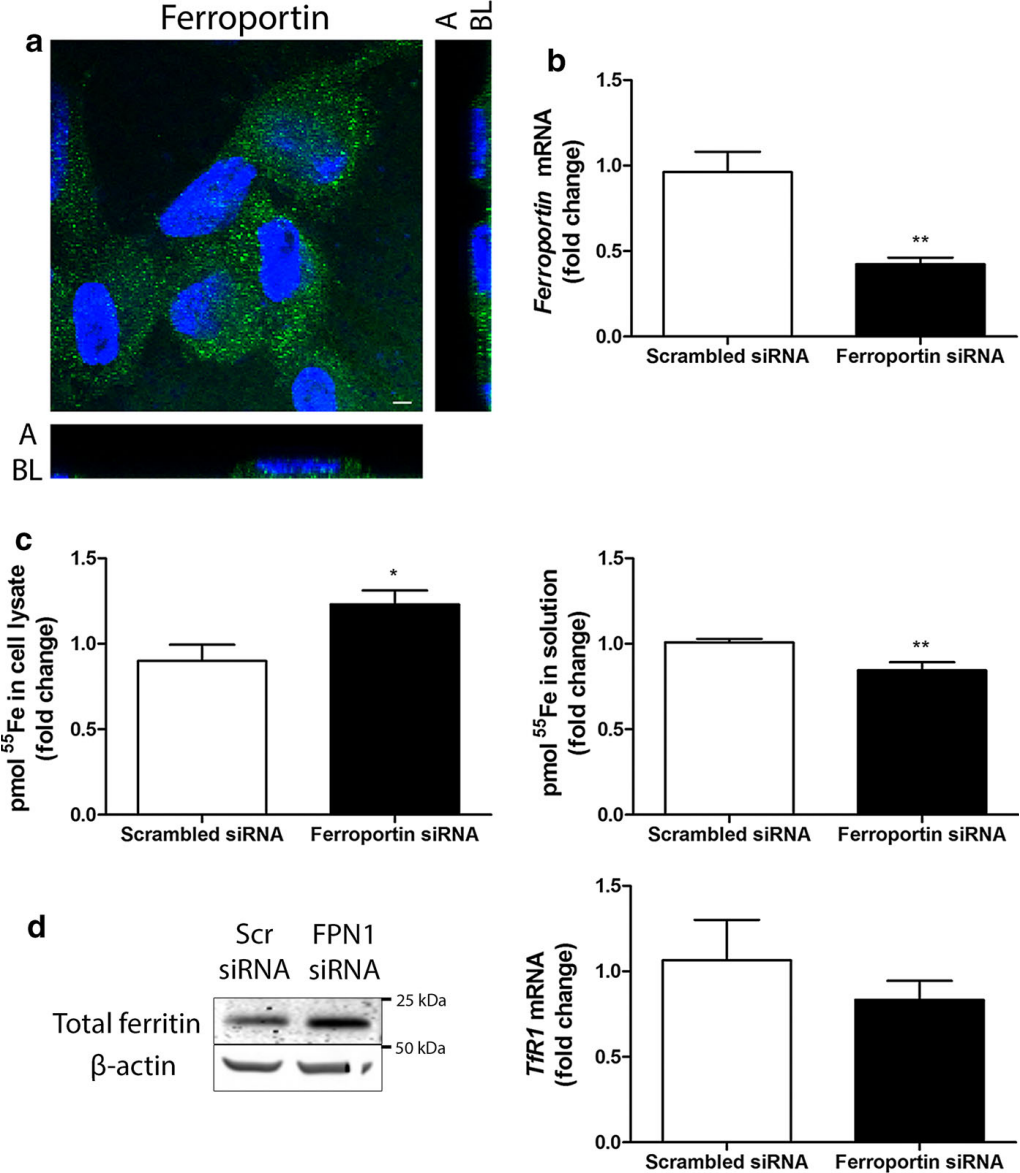
Ferroportin-mediated iron export in ciPTECs. Ferroportin immunostaining (in green) in polarized ciPTECs (**a**). Nuclei counterstained with DAPI (in blue). Confocal images taken in x–y and y–z axis showing apical (A) and basolateral (BL) cellular side. CiPTEC *ferroportin* mRNA expression (**b**), pmol ^55^Fe in cell lysate or solution (**c**), total ferritin protein expression and *transferrin receptor 1* (*TfR1*) mRNA expression (**d**) after transfection with scrambled control (Scr) or ferroportin (FPN1) small interfering RNAs (siRNA). Representative images and graphs showing mean of at least three independent experiments (**a**, n = 3; **b**, n = 9; **c**, n = 4; **d**, both n = 3). Scale bar 5 µM. Student’s t-test was used in **b**, **c** and **d**; *p < 0.05; **p < 0.01. (Color figure online)

We further investigated the role of ferroportin in iron export observed in ciPTECs. Ferroportin immunostaining showed localization near the basolateral cellular side (Fig. 2a), in agreement with its presumed role in iron export in PTs to the plasma (Drakesmith et al. 2015). To study the functional contribution of ferroportin to iron export, we used siRNA technology. Successful ferroportin knockdown was confirmed on mRNA level (p < 0.001, Fig. 2b). Ferroportin knockdown increased cellular ^55^Fe content compared to scrambled control, while ^55^Fe in the exposure solution was decreased (p < 0.05 and p < 0.01, respectively, Fig. 2c). This was complemented by increased total ferritin protein levels and a trend of decreased *TfR1* mRNA levels in ferroportin knockdown compared to control (Fig. 2d). These data confirm that ferroportin functions as an iron exporter in ciPTECs at the basolateral membrane.

### NTBI uptake involves ZIP8 and ZIP14

**Fig. 3:**
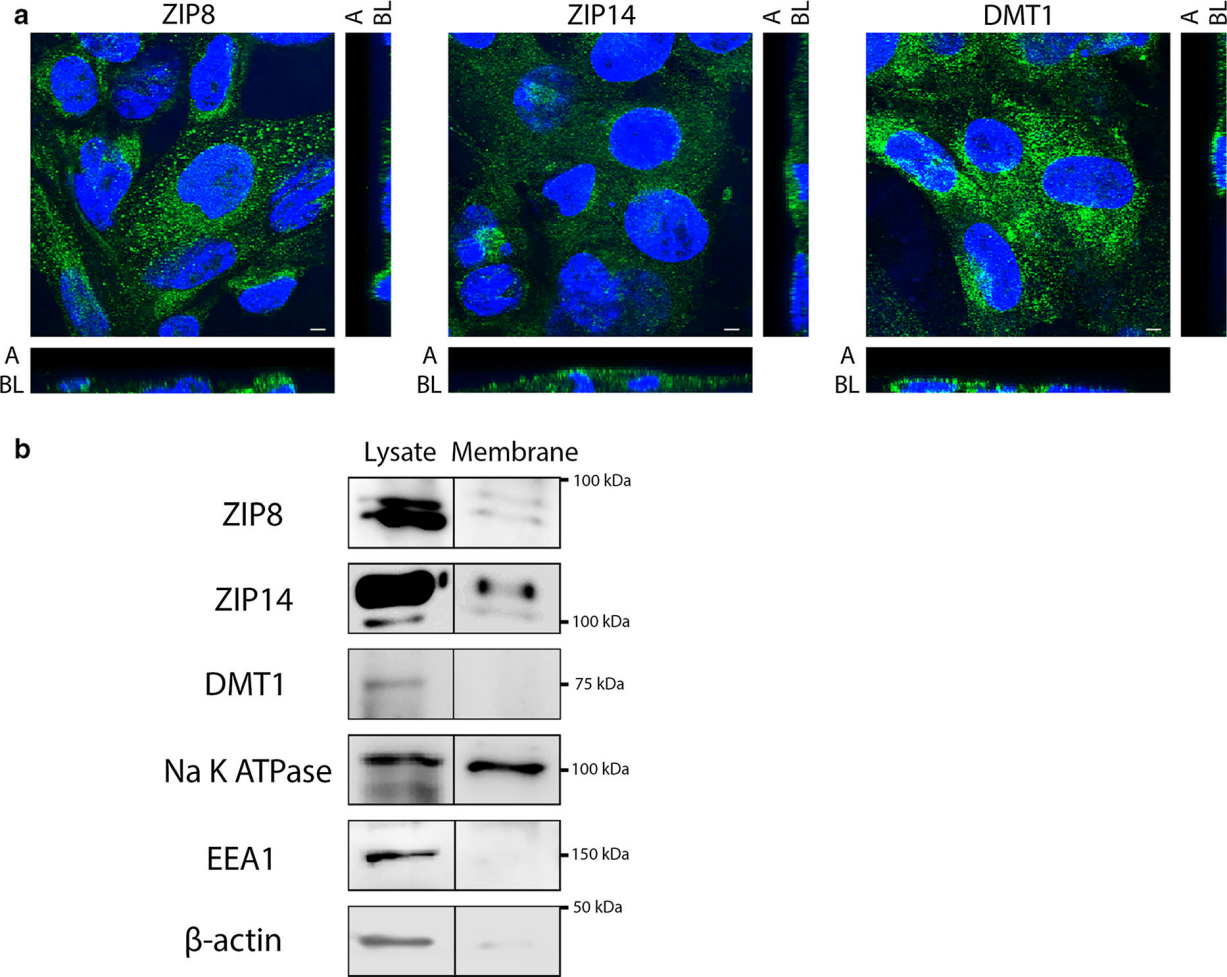
Presence of ZIP8, ZIP14 and DMT1 in ciPTECs. ZIP8, ZIP14 and divalent metal transporter 1 (DMT1) immunostaining (in green) in polarized ciPTECs. Nuclei counterstained with DAPI (in blue). Confocal images taken in x–y and y–z axis showing apical (A) and basolateral (BL) cellular side (**a**). Cell surface biotinylation and immunoblotting of ZIP8, ZIP14 and DMT1 in both membrane fraction and total lysate fraction. In addition, Na K ATPase was used as positive control for cellular membrane proteins, early endosome antigen 1 (EEA1) as negative control for endosomal proteins and β-actin as negative control for cytosolic proteins. Similar Na K ATPase protein levels were loaded in both membrane fraction and total lysate fraction. Prolonged chemiluminescence confirmed depicted findings (data not shown) (**b**). Representative images showing three independent experiments. Scale bar 5 µM. (Color figure online)

To investigate whether ZIP8 and ZIP14 could mediate apical NTBI uptake, we examined their localization in ciPTECs. We found ZIP8 and ZIP14 immunostaining near the apical cellular side in ciPTECs cultured on transwell supports (Fig. 3a). We applied cell surface biotinylation and subsequent immunoblotting to investigate the localisation of these transporters at the plasma membrane and/or in intracellular endosomes. Succesful membrane isolation was confirmed by the presence of plasma membrane marker Na K ATPase in the cell surface fractions, whereas the endosomal marker EEA1 and cytosolic protein β-actin were absent compared to the total cell fractions (Fig. 3b). We detected both ZIP8 and ZIP14 in the ciPTEC plasma membrane and total cell lysate fraction. In contrast, DMT1 immunostaining was detected near the apical membrane, but we did not detect this protein in cell surface fractions (Fig. 3a, b). This indicates ZIP8 and ZIP14 are potential candidates for NTBI uptake at the plasma membrane and subsequent experiments have, therefore, focused on ZIP8 and ZIP14.

**Fig. 4:**
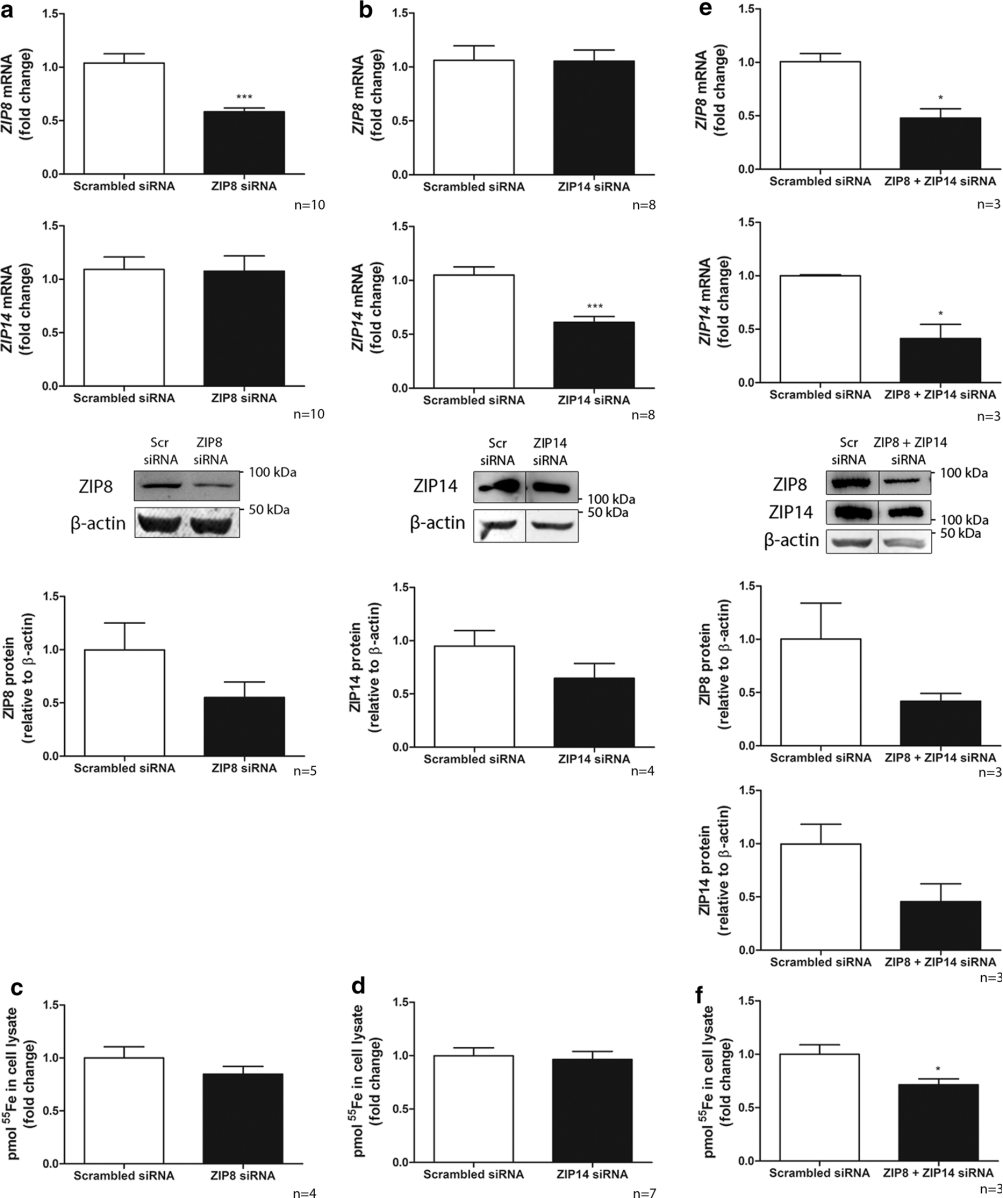
ZIP8 and ZIP14 mediate NTBI uptake in ciPTECs. *ZIP8* and *ZIP14* mRNA and protein expression after transfection with scrambled control (Scr) and either ZIP8 (**a**) or ZIP14 small interfering RNA (siRNA) (**b**). Pmol ^55^Fe uptake in cell lysate after either ZIP8 (**c**) or ZIP14 siRNA transfection (**d**). *ZIP8* and *ZIP14* mRNA and protein expression (**e**) and pmol ^55^Fe uptake in cell lysate (**f**) after transfection with scrambled control or combined ZIP8 + ZIP14 siRNAs. Representative graphs. Number of experiments depicted in each panel. Student’s t-test was used in **a**–**e**; *p < 0.05; ***p < 0.001

We applied ZIP8 and ZIP14 siRNA technology to knockdown each transporter on mRNA level (60% *ZIP8* and 60% *ZIP14* mRNA remaining compared to scrambled control; p < 0.001), without affecting mRNA expression of the other ZIP transporter (*ZIP14* and *ZIP8*, respectively) (Fig. 4a, b). Protein expression was decreased to 50% of scrambled control for ZIP8 and 60% for ZIP14 (Fig. 4a, b). However, silencing of either ZIP8 or ZIP14 did not significantly reduce ^55^Fe uptake (85% and 95% of scrambled control, respectively, Fig. 4c, d). Since both transporters are described to have similar iron transport capacities (Jenkitkasemwong et al. 2012), we also applied combined siRNA knockdown. Both transporters were successfully downregulated on mRNA and protein level (50% *ZIP8* and 40% *ZIP14* mRNA, and 50% ZIP8 and 50% ZIP14 protein remaining, respectively; Fig. 4e). Knockdown of both transporters significantly reduced ^55^Fe uptake (70% of control, p < 0.05, Fig. 4f). This indicates that both ZIP8 and ZIP14 play a role in NTBI uptake and indeed show redundancy in ciPTECs.

### TBI-derived iron uptake involves ZIP14

**Fig. 5:**
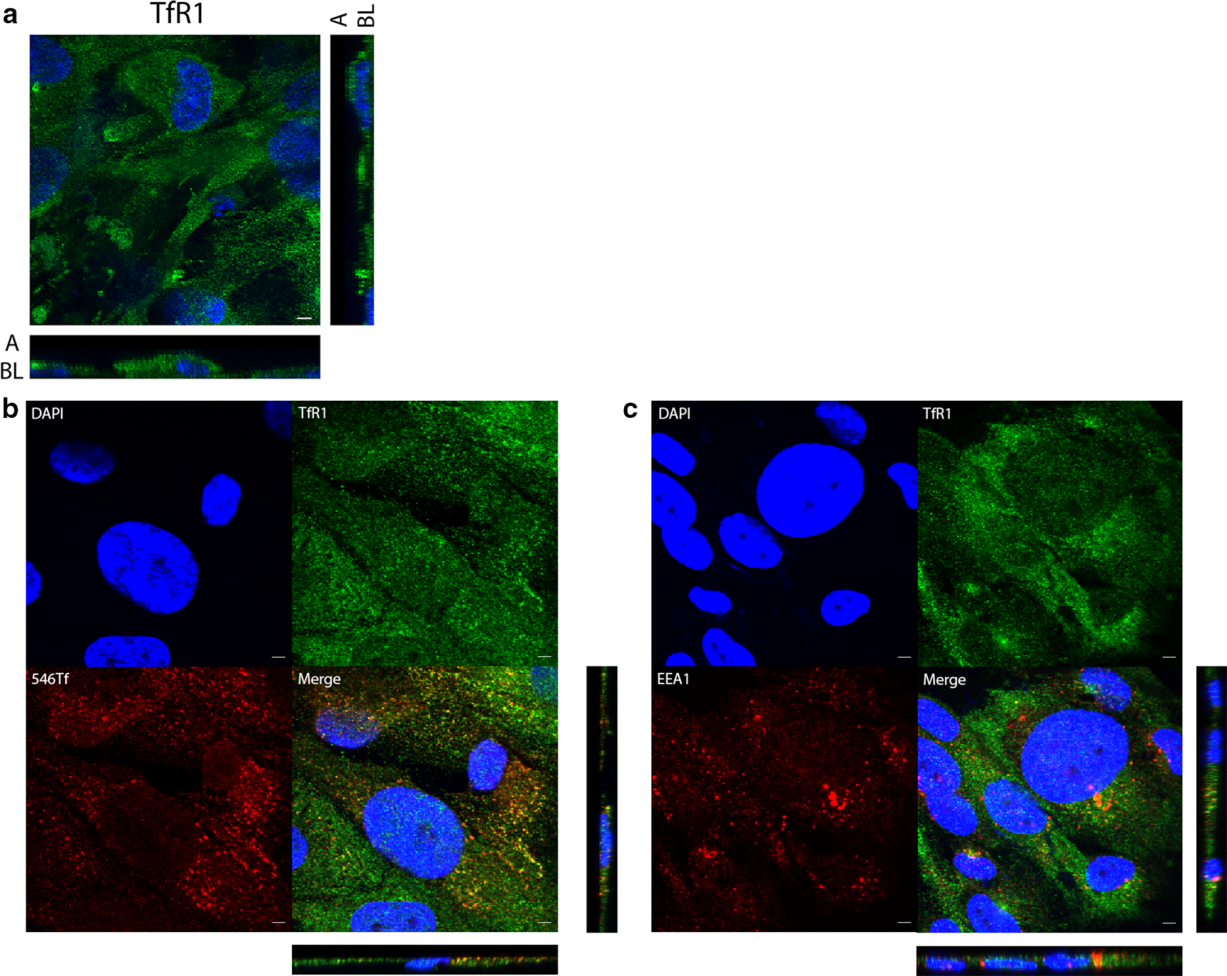
Transferrin receptor 1 mediates Alexa546-Transferrin uptake in ciPTECs. Transferrin receptor 1 (TfR1) immunostaining (in green) in polarized ciPTECs (**a**). TfR1 immunostaining (in green) colocalization with Alexa546-Transferrin (546Tf) internalization (in red) (**b**) or early endosome antigen 1 (EEA1) immunostaining (in red) (**c**). Nuclei counterstained with DAPI (in blue). Confocal images taken in x–y and y–z axis showing apical (A) and basolateral (BL) cellular side. Representative images of three experiments. Scale bar 5 µM. (Color figure online)

**Fig. 6:**
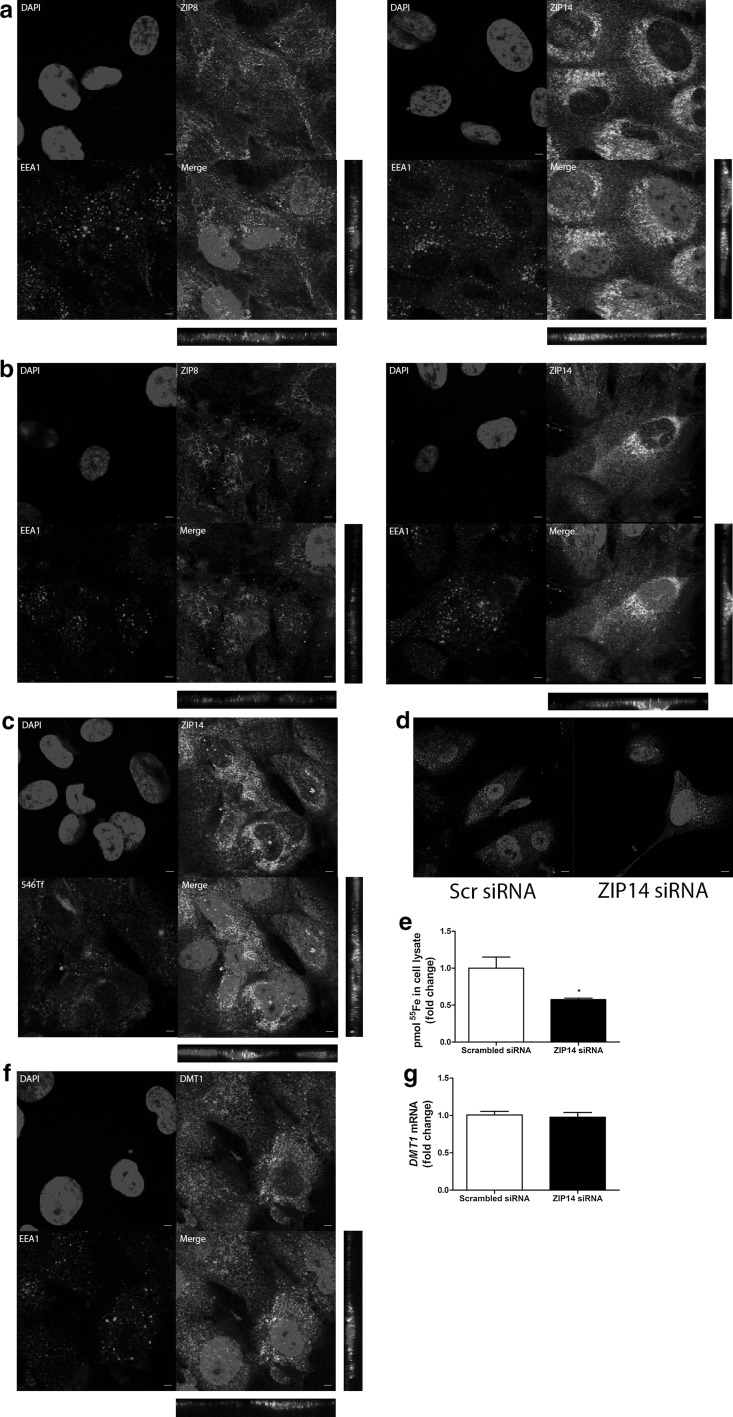
ZIP14 mediates Alexa546-Transferrin uptake in ciPTECs. Double immunostaining of ZIP8 or ZIP14 (in green) and early endosome antigen 1 (EEA1, in red) in unstimulated conditions (**a**) or after 48 h iron overload exposure (**b**). ZIP14 immunostaining (in green) colocalization with Alexa546-Transferrin (546Tf) internalization (in red) (**c**). 546Tf internalization (**d**) or pmol ^55^Fe in cell lysate after ^55^Fe-TBI exposure (**e**) after transfection with scrambled control or ZIP14 small interfering RNA (siRNA). Double immunostaining of divalent metal transporter 1 (DMT1, in green) and EEA1 (in red) (**f**). *DMT1* mRNA expression (**g**) after transfection with ZIP14 or scrambled control siRNAs. Nuclei counterstained with DAPI (in blue). Confocal images show x–y and y–z axis. Representative images and graphs of three experiments. Scale bar 5 µM. Student’s t-test was used in **e**, **g**; *p < 0.05. (Color figure online)

We detected TfR1 near the ciPTEC apical membrane using fluorescent immunostaining (Fig. 5a) and found TfR1 to colocalize with both EEA1 and Alexa546-Transferrin (Fig. 5b, c), indicating TBI uptake involves TfR1 via endocytosis. Subsequently, we examined whether ZIP8 and ZIP14 may play a role in iron uptake resulting from TBI endocytosis. We found only ZIP14, but not ZIP8, to colocalize with EEA1 (Fig. 6a), suggesting a possible involvement of ZIP14 in transport of iron out of the endosome towards the cytosol. This staining pattern was not affected by iron exposure (Fig. 6b), indicating ZIP8 is absent from ciPTEC endosomes independent of cellular iron status. ZIP14 immunostaining colocalized with Alexa546-Transferrin uptake (Fig. 6c), but ZIP14 knockdown did not affect Alexa546-Transferrin uptake (Fig. 6d). Cellular ^55^Fe content, however, was decreased in ZIP14 knockdown cells compared to control after ^55^Fe-TBI exposure (p < 0.05, Fig. 6e). This indicates ZIP14 could be involved in TBI-derived iron uptake. DMT1 was also found to colocalize with EEA1 immunostaining (Fig. 6f) and, even though *DMT1* mRNA expression did not change with ZIP14 knockdown (Fig. 6g), ZIP14 and DMT1 could be redundant in TBI-derived iron uptake.

## Discussion

**Fig. 7:**
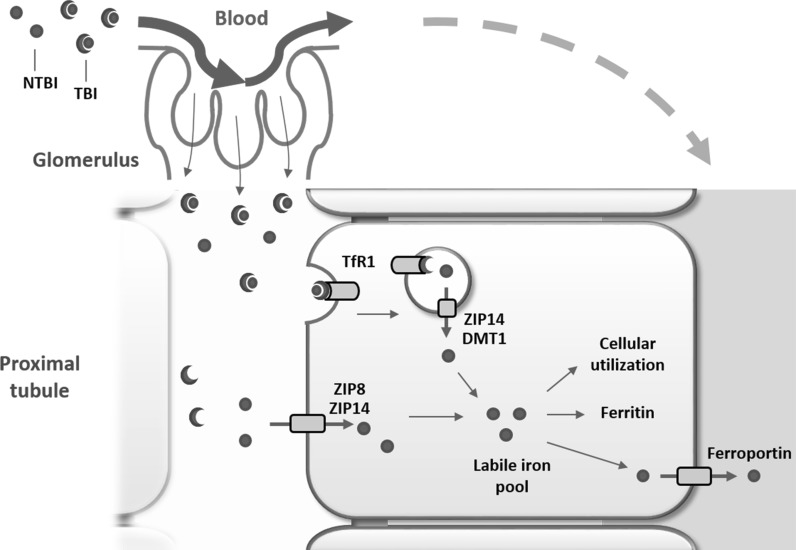
Proposed NTBI and TBI handling in proximal tubular epithelial cells. Non-transferrin-bound iron (NTBI) and transferrin-bound iron (TBI) present in the systemic circulation are filtered by the glomerulus into the tubular lumen and are subsequently reabsorbed by proximal tubular cells. NTBI uptake at the plasma membrane involves both ZIP8 and ZIP14 that show redundancy. TBI uptake involves TfR1-mediated endocytosis. Subsequently, iron is transported from the endosome towards the cytosol via ZIP14 and potentially also divalent metal transporter 1 (DMT1). Once in the cytosol, in the labile iron pool, iron is utilized, stored in ferritin or exported back into the circulation by ferroportin at the basolateral membrane. (Color figure online)

Evidence of renal complications in patients with systemic iron overload is accumulating, while the molecular mechanisms of renal NTBI and TBI handling in systemic iron overload remain unclear. We have shown that human PTs take up both NTBI and TBI. NTBI uptake involves both ZIP8 and ZIP14, which demonstrate redundancy. In contrast, TBI uptake is mediated by TfR1 endocytosis, where ZIP14, but not ZIP8, may be involved in iron release from the endosome into the cytosol (Fig. 7).

In addition to studies that report the suitability of ciPTECs to study renal physiology (Jansen et al. 2014; Wilmer et al. 2010), we have demonstrated that this model is also qualified to study renal PT iron handling as it contains mechanisms for iron uptake, intracellular handling and export. Using this model, we newly demonstrated the subcellular distribution of endogenous ZIP8 and ZIP14 in human PTs using cell surface biotinylation, immunoblotting and immunofluorescent colocalization stainings, which extends previous studies that mostly relied on overexpression models (Thevenod and Wolff 2016). Our results show variability in divalent iron transporter localization in PT cells in terms of their localization at the plasma membrane or at the endosomal membrane. NTBI exposure was experimentally approached by addition of FeC to serum-free medium, preventing TBI formation, but allowing formation of citrate-bound NTBI. Interestingly, we observed a difference in involvement of ZIP8 and ZIP14 in NTBI and TBI uptake by ciPTECs. Although siRNA silencing in ciPTECs was limited, i.e. silencing of ~ 50% was achieved for all examined proteins, including ZIP8, ZIP14, ferroportin and ZIP8 and ZIP14 combined, it nevertheless demonstrated that both ZIP8 and ZIP14 were involved in NTBI uptake, whereas only ZIP14 may play a role in TBI-derived iron uptake. These observations support the notion that we studied uptake of two distinct iron species. ZIP8 and ZIP14 were both present at the ciPTEC plasma membrane and showed redundancy in tubular NTBI import. This redundancy for plasma membrane NTBI uptake is likely since both ZIP8 and ZIP14 are reported to transport iron together with HCO_3_^−^ at pH 6.5–7.5 (He et al. 2006; Pinilla-Tenas et al. 2011; Wang et al. 2012; Zhao et al. 2010). Also in hereditary hemochromatosis mouse models lacking ZIP14, NTBI uptake in the kidney was still detected, further corroborating redundancy of iron transporters (Jenkitkasemwong et al. 2015). Besides ZIP8 and ZIP14, other mechanisms for NTBI uptake have been described in various organs, which could explain why even the combined silencing of ZIP8 and ZIP14 did not abolish NTBI uptake in our model. In human hepatocytes, DMT1 was detected at the plasma membrane and was found to mediate NTBI uptake in these cells (Shindo et al. 2006). Although we previously detected DMT1 in human PTs (van Raaij et al. 2018), it was not present at the plasma membrane in ciPTECs. As a result, we believed that a role for DMT1 in direct NTBI uptake at the plasma membrane was unlikely and focussed our studies on ZIP8 and ZIP14. Furthermore, in the circulation, NTBI can be bound to albumin (Silva and Hider 2009) and, as such, be reabsorbed in the PT by the multiligand megalin–cubilin receptor complex (Christensen et al. 2012). Alternatively, L-type voltage-dependent calcium channels have been reported to facilitate NTBI uptake in cardiomyocytes (Oudit et al. 2003; Oudit et al. 2006), which may also be the case in the human kidney, as these channels have been demonstrated in rat kidney (Zhao et al. 2002).

We showed that TBI is internalized predominantly from the apical cellular side and this is mediated by the endocytic transporter TfR1. In addition, also the megalin–cubilin receptor complex is reported to take up TBI in PTs (Kozyraki et al. 2001). The fact that TBI uptake was reduced during iron loading implies IRE-IRP-regulated restriction of TBI uptake, hence suggesting that TBI uptake in PTs is predominantly mediated through TfR1. Moreover, TfR1 reduction in iron excess conditions suggests that TBI uptake is not the major cause of PT iron accumulation during iron overload. Since ZIP8 and ZIP14 abundance is not controlled by IRE-IRP regulation (Jenkitkasemwong et al. 2012), it is plausible that especially unrestricted tubular NTBI uptake by these proteins results in renal iron loading and potentially subsequent iron-mediated kidney injury during systemic iron overload.

In ciPTECs, ZIP14 may play a role in transport of iron from the endosome towards the cytosol. In the endosome, ferric iron is released from transferrin, and, subsequently, reduced to ferrous iron, possibly by a STEAP protein or Prion protein, which have been found in human and mouse kidney, respectively (Ohgami et al. 2005, 2006; Tripathi et al. 2015). Iron is found to dissociate from transferrin in early endosomes at pH 6.5 (Nunez et al. 1990), and ZIP14 is shown to transport iron at a similar pH (Pinilla-Tenas et al. 2011; Zhao et al. 2010). Furthermore, ZIP14 is shown to mediate iron uptake from TBI in hepatocytes (Zhao et al. 2010), while both ZIP8 and ZIP14 are found to mediate iron uptake from TBI uptake in neurons (Ji and Kosman 2015). Our results support the novel conclusion that ZIP14, but not ZIP8, may be involved in TBI-derived iron uptake in PT early endosomes. Our data do not substantiate the presence of ZIP8 in endosomes, which was shown for overexpressed rat ZIP8 in HEK293 cells (Wang et al. 2012). However, TBI-derived iron uptake was not abolished with ZIP14 knockdown. Indeed, renal iron loading was observed in several hereditary hemochromatosis mouse models with TBI exposure despite ZIP14 knockout (Jenkitkasemwong et al. 2015). Besides ZIP14, we detected DMT1 in endosomes in ciPTECs and, therefore, DMT1 is a potential candidate for endocytic iron transport in PTs, like is reported in hepatocytes (Gunshin et al. 1997; Tabuchi et al. 2000; Wang and Knutson 2013). DMT1 is shown to transport iron at pH 5.5 (Gunshin et al. 1997). Therefore, it is possible that DMT1 transports iron into the cytosol from late endosomes, which are characterized by a pH between 4.5 and 5.5 (Pinilla-Tenas et al. 2011), in PTs, and this is an interesting topic for future studies (in ciPTECs). Moreover, also TRPML1 (mucolipin1) is reported to transport iron in late endosomes or early lysosomes (Dong et al. 2008). The importance of this transporter in endosomal iron uptake is illustrated by findings of decreased cytosolic iron levels in TRPML1 knockout fibroblasts, and iron-deficiency and anaemia in patients with TRPML1 mutations (Altarescu et al. 2002; Dong et al. 2008). TRPML1 has also been found in the kidney and could, thus, mediate endosomal iron transport into the cytosol (Cheng et al. 2010; Philpott et al. 2017; Thevenod and Wolff 2016).

Although ZIP8 and ZIP14 demonstrated redundancy in ciPTEC NTBI transport, differential functions for both transporters have been reported. Despite similar iron transport capacities (Jenkitkasemwong et al. 2012), ZIP8 and ZIP14 show a different localization pattern at the organ level, suggesting that these transporters have different roles in NTBI uptake from the circulation. While ZIP14 has been reported as the main NTBI uptake transporter in the liver and pancreas (Jenkitkasemwong et al. 2015), ZIP8 is implicated to be the major NTBI transporter in hippocampal neurons (Ji and Kosman 2015). As such, mutations in ZIP8 and ZIP14 in patients both lead to changes in metal transport, but show distinct phenotypes (Boycott et al. 2015; Li et al. 2016; Park et al. 2015; Tuschl et al. 2016). In addition to PTs, ZIP8 and ZIP14 are also present in human distal tubular epithelial cells (Ajjimaporn et al. 2012; van Raaij et al. 2018), but it remains to be investigated to what extent both transporters are involved in NTBI handling in the distal nephron.

In our current study in human ciPTECs, we observed ferroportin expression at the basolateral side of the cell, whereas expression at the apical and basolateral membrane was reported previously in murine PT (Moulouel et al. 2013; Starzynski et al. 2013; Veuthey et al. 2008; Wolff et al. 2011; Zarjou et al. 2013). We recently showed ferroportin expression in human kidney biopsies also at the basolateral cellular side (van Raaij et al. 2018). Moreover, our findings in ciPTECs show that ferroportin functioned solely as iron exporter, and not as iron importer as has previously been suggested (Zarjou et al. 2013). Our results are in line with the general belief of a ferroportin cellular iron export function (Nemeth et al. 2004). Since to date no other iron cellular iron exporters have been identified, ferroportin is essential for physiological renal iron handling. Moreover, reabsorption of filtered iron and export back into the systemic circulation is crucial for maintaining adequate body iron levels as well as preventing intracellular renal iron loading and potential iron-induced renal toxicity.

In summary, our findings in human ciPTECs show that ZIP8 and ZIP14 are involved in TBI and NTBI uptake. Nevertheless, the results also demonstrate high redundancy in divalent metal transport in ciPTECs, suggesting complex mechanisms of iron uptake. Future studies should be aimed at elucidating the apparent complex process of proximal tubular iron reabsorption to further assess the contribution of individual transporters to iron uptake in physiological conditions and during systemic iron overload.

## Electronic supplementary material

Below is the link to the electronic supplementary material.

Supplementary material 1 (PDF 432 kb)
