# Supplementary material for: Iron uptake by ZIP8 and ZIP14 in human proximal tubular epithelial cells
Source: Biometals. 2019 Feb 26;32(2):211–26. doi: 10.1007/s10534-019-00183-7 (PMC6437295; doi:10.1007/s10534-019-00183-7)
Supplement: Supplementary file 1 — Supplementary material 1 (PDF 432 kb) [file 10534_2019_183_MOESM1_ESM.pdf]

Online Resources

Iron uptake by ZIP8 and ZIP14 in human proximal tubular epithelial cells

SEG van Raaij MSc[a], SKS Srai PhD[b], DW Swinkels MD, PhD[a], RPL van Swelm PhD[a*]

[a]Department of Laboratory Medicine, Radboud Institute for Molecular Life Sciences, Radboud university medical center, Nijmegen, the Netherlands; [b]Department of Structural & Molecular Biology, Division of Biosciences, University College London, London, United Kingdom; [*]corresponding author

Corresponding author: RPL van Swelm, PhD, Rachel.vanSwelm@Radboudumc.nl

Online Resource 1: Overview used primary antibodies

Protein	Primary antibody
TfR1	Thermo Fisher Scientific 136800, 1:500
ZIP8	ProteinTech 20459-1-AP, 1:500
ZIP14	Atlas Antibodies HPA016508, 1:500
DMT1	Abcam ab55735, 1:350
ZO-1	Invitrogen 402200, 1:100
EEA1	Abcam ab2900, 1:1000 (immunoblot and immunostaining with DMT1)<br>Abcam ab70521, 1:200 (immunostaining with TfR1, ZIP8, ZIP14)
Na K ATPase	Koenderink lab, 1:2500 (Koenderink et al. 2003)
Total ferritin	Sigma Aldrich F5012, 1:250
Ferroportin	Abcam ab85370, 1:100
β-actin	Sigma Aldrich A5441, 1:100,000

DMT1, divalent metal transporter 1; EEA1, early endosome antigen 1; TfR1, transferrin receptor 1; ZO-1, Zona occludens 1.

Online Resource 2: Overview used quantitative PCR primers

Protein	Forward primer (5'- 3')	Reverse primer (5'- 3')
TfR1	CCCAGTTGCTGTCCTGATATAGA	TTGAGAAAACAATGCAAAATGTG
ZIP8	TTATCTCTGTCCCCCTTTGTCCTC	CGCCTTCGAAAGAACAGCAG
ZIP14	CTGGACCACATGATTCCTCAG	GAGTAGCGGACACCTTCCAG
DMT1	ATGGCCCTCACATTTGGATA	CGAACATGCCCTTGAGTACC
Ferroportin	GGGGTCGCCTAGTGTCAT	CAGGTAGTCGGCCAAGGAT
HPRT	GCTATAAATTCTTTGCTGACCTGCTG	AATTACTTTTATGTCCCCTGTTGACTGG

DMT1, divalent metal transporter 1; HPRT, Hypoxanthine-guanine phosphoribosyltransferase; TfR1, transferrin receptor 1.

References

Koenderink JB, Geibel S, Grabsch E, De Pont JJ, Bamberg E, Friedrich T (2003) Electrophysiological analysis of the mutated Na,K-ATPase cation binding pocket The Journal of biological chemistry 278:51213-51222 doi:10.1074/jbc.M306384200
